# Survey of moniliformin in wheat- and corn-based products using a straightforward analytical method

**DOI:** 10.1007/s12550-017-0287-9

**Published:** 2017-08-08

**Authors:** Marta Herrera, Ruud van Dam, Martien Spanjer, Joyce de Stoppelaar, Hans Mol, Monique de Nijs, Patricia López

**Affiliations:** 1 Instituto Agroalimentario de Aragón IA2, Veterinary Faculty, Universidad de Zaragoza-CITA, 50013 Zaragoza, Spain; 2 RIKILT Wageningen University & Research, Akkermaalsbos 2, 6708 WB Wageningen, The Netherlands; 3 NVWA—Netherlands Food and Consumer Product Safety Authority, Catharijnesingel 59, 3511 GG Utrecht, The Netherlands

**Keywords:** Moniliformin, LC-MS/MS, LC-Orbitrap-HRMS, Maize, Wheat

## Abstract

A straightforward analytical method was developed and validated to determine the mycotoxin moniliformin in cereal-based foods. Moniliformin is extracted with water and quantified with liquid chromatography tandem mass spectrometry, and its presence confirmed with liquid chromatography-Orbitrap-high-resolution mass spectrometry. The method was validated for flour, bread, pasta and maize samples in terms of linearity, matrix effect, recovery, repeatability and limit of quantification. Quantification was conducted by matrix-matched calibration. Positive samples were confirmed by standard addition. Recovery ranged from 77 to 114% and repeatability from 1 to 14%. The limit of quantification, defined as the lowest concentration tested at which the validation criteria of recovery and repeatability were fulfilled, was 10 μg/kg. The method was applied to 102 cereal-based food samples collected in the Netherlands and Germany. Moniliformin was not detected in bread samples. One of 22 flour samples contained moniliformin at 10.6 μg/kg. Moniliformin occurred in seven out of 25 pasta samples at levels around 10 μg/kg. Moniliformin (MON) was present in eight out of 23 maize products at levels ranging from 12 to 207 μg/kg.

**Electronic supplementary material:**

The online version of this article (doi:10.1007/s12550-017-0287-9) contains supplementary material, which is available to authorized users.

## Introduction

**Fig. 1:**
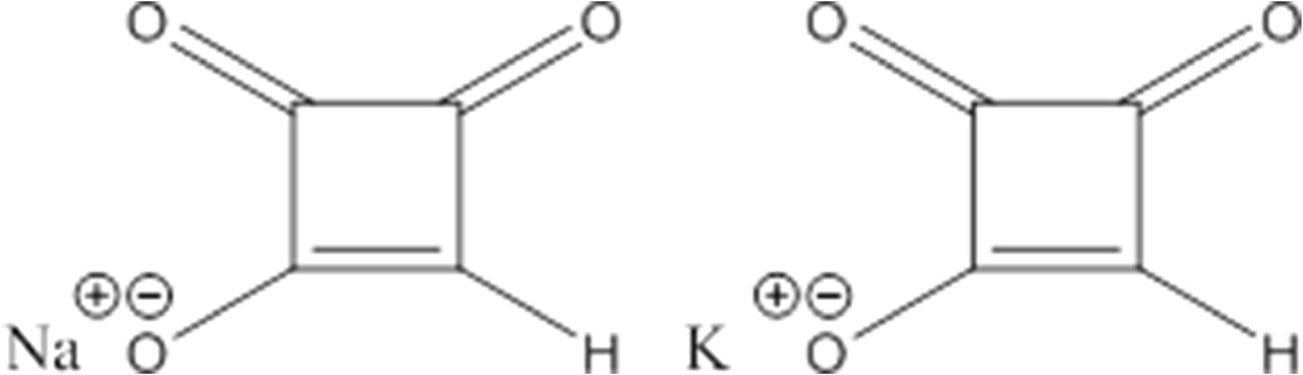
Sodium and potassium salt structures of moniliformin

Moniliformin (MON) is a frequently worldwide occurring mycotoxin in cereals and is produced by many *Fusarium* species (Uhlig et al. 2004), including *Fusarium avenaceum*, *Fusarium proliferatum*, *Fusarium subglutinans*, *Fusarium tricinctum* and *Fusarium verticillioides*. MON is a small (98.0081 g/mol), highly polar, acidic molecule. Because of the low pKa (0.5–1.7) of the free acid, it does not occur as acid in nature but as water-soluble sodium or potassium salt (Fig. 1). It was first discovered in 1973 by Cole et al. (1973) and characterized in 1974 by Springer et al. (1974).

The toxicity of MON has been studied in in vitro and in vivo conditions. MON inhibits, in vitro, multiple enzyme systems such as pyruvate dehydrogenase, transketolase, aldose reductase, glutathione peroxidase and glutathione reductase. MON has an acute toxicity comparable to T-2 toxin in ducklings and chickens with LD_50_ varying from 3.7 to 5.4 mg/kg body weight (Jestoi 2008). Peltonen et al. (2010) applied a No Observed Adverse Effect Level (NOAEL) of 10 mg/kg bw/day based on subchronic 28-day animal experiments to deduce a provisional tolerable daily intake of 0.1 mg/kg bw/day. Currently, there is no legislation on MON in the EU, but the European Food Safety Authority (EFSA) is currently assessing its risks to public health.

MON has been detected in various cereal commodities worldwide (Jestoi 2008) at different concentration levels. MON occurred in Norwegian grains at incidences of 31.5% in oats, 76% in wheat and 25% in barley (Uhlig et al. 2004), with a limit of detection (LOD) of 40 μg/kg. The range of MON occurrence in oats, wheat and barley was LOD—210, LOD—950 and LOD—380 μg/kg, respectively. MON was detected in 93% of naturally contaminated maize in Italy (LOD—2606 μg/kg), with a LOD of 1 μg/kg (Scarpino et al. 2013). A small survey on the occurrence of MON in cereal samples (corn, wheat, rye and oat kernels and flours) in Germany with a LOD of 0.7 μg/kg showed that 20 out of 23 samples were contaminated in the range LOD—126 μg/kg (von Bargen et al. 2012).

There is limited information on the fate of MON during food processing. MON has been reported to be stable in food processes that occur under neutral (baking of maize bread) or acidic (wet milling of maize) conditions (Pineda-Valdes et al. 2003) but to degrade around 70% under alkaline cooking (Pineda-Valdes et al. 2002). Due to the hydrophilic character of MON, it is expected that cooking of MON-containing dry pasta in water will reduce MON content of cooked pasta via leaching of MON to the cooking water.

Several analytical methods for the quantification of MON have been reported so far: thin-layer chromatography (TLC) (Jansen and Dose 1984), ion-pair high-performance liquid chromatography (HPLC) with UV detection (Sharman et al. 1991), fluorescence detection (Filek and Lindner 1996) or detection by atmospheric pressure chemical ionisation mass spectrometry (APCI-MS) (Sewram et al. 1999), ion chromatography (IC) (Kandler et al. 2002), capillary electrophoresis coupled to diode array detector (CE-DAD) and gas chromatography-mass spectrometry after derivatization (GC-MS) (Gilbert et al. 1986). The reported limits of quantification (LOQ) were 24 μg/kg for HPLC-APCI-MS with derivatisation, 120 μg/kg for ion chromatography or 20 μg/kg for GC-MS or HPLC-FLD. Because of its ionic nature, MON is weakly retained by reversed-phase chromatography (RPLC). Nevertheless, RPLC is used in routine methods either with the formation of ion pairs (Sewram et al. 1999) or employing polar silica columns such as Gemini, where the phenyl-hexyl-bonded phase showed better retention compared to that of C_18_ (Lim et al. 2015; von Bargen et al. 2012). Hydrophilic interaction chromatography (HILIC) has been used to achieve good chromatographic separation of MON, but it suffered from poor peak shape (Scarpino et al. 2013). Recently, a new type of columns, so-called “mix-mode”, that combine separation based on anion exchange and hydrophobic interactions has been successfully applied (Hallas-Moller et al. 2016).

LC-MS/MS is currently the preferred method for mycotoxin analysis. MON has also been included in the scope of multi-methods for mycotoxin analysis by LC-MS/MS (Sulyok et al. 2007; Varga et al. 2013). The reported LOQ of the multi-method by Sulyok et al. was 20 μg/kg (calculated as spiked samples with a signal-to-noise ratio of 10:1), while the LOQ of the multi-method for nuts by Varga et al. was around 5 μg/kg, based on the signal-to-noise ratio of a matrix-matched standard. The drawback of LC-MS/MS methods is that only one ion transition (97 > 41) can be monitored, thus not fulfilling Commission Decision 2002/657/EC, which requires at least two product ions for a reliable identification (EC 2002). The application of high-resolution MS (HRMS) fulfils the EU identification criteria (Hallas-Moller et al. 2016; Lim et al. 2015; von Bargen et al. 2012), adding an extra identification point with the accurate mass of the compound.

The aim of this study was the development and validation of an analytical method combining LC-MS/MS (quantification) and LC-Orbitrap-HRMS (confirmation) to determine MON in bread, dry pasta, wheat flour and maize products with a limit of quantification of 10 μg/kg.

## Materials and methods

### Chemicals and reagents

Moniliformin sodium salt was purchased from Sigma-Aldrich (Zwijndrecht, the Netherlands). A standard stock solution of 100 μg/mL was prepared in acetonitrile/water (9/1 v/v) and kept at 4 °C for a maximum of 6 months. Working solutions for method development, validation and sample analysis were prepared from this stock solution.

Acetonitrile and methanol, both UPLC grade, were purchased from Actu-all Chemicals (Oss, the Netherlands). Formic acid (98–100%) and acetic acid (99%) were supplied by Merck (Amsterdam, the Netherlands), magnesium sulphate by Sigma-Aldrich and ammonium formate by Acros Organics (Thermo Fisher Scientific, Geel, Belgium).

Strata® SAX SPE columns were supplied by Phenomenex (Utrecht, the Netherlands) and MycoSep® 240 MON by Romer Labs (Food Risk Management, Oostvoorne, the Netherlands).

### Samples

A total of 102 cereal-based food samples were purchased from retail stores in the Netherlands and Germany during 2016 and 2017. The list of samples was composed of 32 bread-type samples, 25 dry pasta samples, 22 flour samples and 23 maize products. Samples were randomly collected and from mostly big nationwide discounters.

Samples of dry pasta, bread and corn flakes were ground at ambient temperature. Samples were stored at −18 °C until processing. For further details on samples, see Supplementary Information SS1.

### Optimisation of the chromatographic separation

The following chromatographic columns were assessed: Synchronis™ HILIC 100 × 2.1 mm 1.7 μm (Thermo Fisher Scientific, Waltham, MA, USA), Gemini® C6-Phenyl 100 × 2.0 mm 3 μm (Phenomenex, Utrecht, the Netherlands) and SeQuant® ZIC®-HILIC 100 × 2.1 mm 5 μm (Merck Millipore, Darmstadt, Germany).

Water with 1% formic acid and water buffered at pH 6.4 with ammonium formate were tested as mobile phase A, while acetonitrile (with and without formic acid 1%) and methanol with 1% formic acid were used as mobile phase B.

Optimization was carried out in an Acquity UPLC coupled with a Xevo TQ-S mass spectrometer (Waters, Milford, MA, USA).

### Optimisation of the extraction procedure

Most of the analytical methods reported in literature for the analysis of MON comprise an extraction with acetonitrile/water (84:16, v/v) and further purification of the extract with SAX-like SPE columns (von Bargen et al. 2012) or MycoSep® MON 240 (Lim et al. 2015; Scarpino et al. 2013). Extraction with acetonitrile/water (84:16, v/v) has been reported to provide the best MON recoveries from maize (Parich et al. 2003).

Different extraction procedures of MON from bread were tested in this study: (a) acetonitrile/water (84:16, v/v), (b) acetonitrile/water with 1% formic acid (84:16, v/v), (c) acetonitrile/water with 1% acetic acid (84:16, v/v), (d) QuEChERS extraction with acetonitrile/water with 1% acetic acid (84:16, v/v) plus clean-up with 4 g of magnesium sulphate and 1 g of sodium acetate (Lopez et al. 2016), (e) acetonitrile/water (50:50 v/v) and (f) water 100%.

The performance of the clean-up procedures with SAX-Strata and MycoSep® MON 240 was also studied. The protocol with SAX was described elsewhere (von Bargen et al. 2012) and, briefly, consisted of activating the SAX column by adding consecutively 2 mL of methanol, 2 mL of water and 2 mL of 0.1 M HCl before applying the dissolved sample extract. Subsequently, matrix constituents were removed from the column with 2 mL of methanol/water (50:50, v/v) followed by 2 mL 0.1 M HCl. MON was eluted with 2 mL of 1 M HCl, and the extract was evaporated to dryness at 40 °C under a stream of nitrogen. The dried residue was reconstituted in 500 μL of methanol/water (50:50, v/v).

The clean-up procedure with MycoSep® MON 240 was adapted from that described by the supplier (Romer Labs®). MycoSep® 240 MON columns were pushed into test tubes containing 5 mL of extracts. The interferences are retained by the columns resulting in clean extracts, which were injected in the LC-MS/MS system. Additionally, the cleaned extracts were evaporated under a gentle nitrogen stream either down to 0.5 mL or to dryness. The extracts that were evaporated till dryness were reconstituted with 500 μL of methanol/water (50:50, v/v).

The extraction and clean-up protocols were tested on bread spiked with 50 and 100 μg/kg of MON.

### Final experimental procedure

An aliquot of 2.5 g sample was extracted with 10 mL Milli-Q water for 1 h head-over-head and then centrifuged for 10 min at 2600 ×*g*. An aliquot of 1.5 mL supernatant was transferred into an Eppendorf® and further centrifuged for 10 min at 11,500 ×*g*. Next, 500 μL extract was pipetted in mini-uniprep PTFE filter vials and filtered before analysis by LC-MS/MS or LC-HRMS.

Samples were first analysed and quantified in an Acquity UPLC coupled with a Sciex QTRAP® 6500 mass spectrometer (AB Sciex LLC, Framingham, MA, USA). Chromatographic separation was carried out by injecting 10 μL extract into a Synchronis™ HILIC column (100 × 2.1 mm, 1.7 μm). The mobile phases were 100 mM ammonium formate in water pH 6.4 (A) and acetonitrile (B). The flow rate was set at 0.2 mL/min. Gradient elution was applied as follows: the initial composition of 10% A was kept for 2 min, raised up to 90% in 6 min, kept for 5 min and then set back in 2 min to its initial settings, which were kept for 5 min to re-equilibrate the column. The analysis of the samples was carried out in electrospray negative ionisation mode, and the monitored transition was 97 > 41.3 with the following settings: declustering potential (DP) −30 V, entrance potential (EP) −10 V, collision energy (CE) −30 V and collision cell exit potential (CXP) −10 V. The other settings related to the ionisation of extracts were curtain gas (CUR) 20 psi, collisional activated dissociation gas (CAD) medium, source temperature 400 °C, ion spray voltage −4000 V, nebulizer gas (GAS1) 60 psi, heater gas (GAS2) 60 psi and additional temperature 400 °C. The system was controlled using the software packages Analyst® and MultiQuant 2.1.1 (Sciex).

Confirmation of the positive samples was performed on an Ultimate 3000 UHPLC system consisting of a quaternary pump, an autosampler and a column oven, coupled by a HESI-II electrospray source to a Q-Exactive Orbitrap™-based mass spectrometer (Thermo Fisher Scientific). The HESI-II electrospray source was operated with the following parameters recommended by the MS software for the LC flow rate used: capp. voltage −2.5 kV; sheath gas 45 AU; auxiliary gas 10 AU; cone gas 2.20 AU; capillary temperature 250 °C; heater temperature 400 °C. Extracts were measured with one full-scan even (mass range 96–98 m/z) with a resolving power of 140,000. The automatic gain control (AGC) was set at 5 × 10^6^ and the maximum inject time at 500 ms. The system was controlled using the software packages Xcalibur 3.0, Chromeleon MS Link 2.14 and Q-Exactive Tune 2.3 (Thermo Scientific).

### Method validation

The method was validated in terms of linearity, matrix effects, repeatability, recovery and limit of quantification (LOQ).

Linearity was evaluated in the range of 2.5–25 ng/mL standards in matrix which corresponded to 10–100 μg/kg in sample. Matrix-matched calibration lines were prepared in bread, pasta, wheat flour and corn flour. The method was considered linear when the correlation coefficient of the regression line was higher than 0.99, and the back-calculated concentrations should not exceed 30% of the nominal value.

Matrix effects can enhance or suppress the instrumental signal that entails bias results. Matrix effects were assessed by comparing the response of the standards prepared in solvent with that of the standards prepared in matrix.

The recovery and repeatability were calculated in six-fold for samples spiked at 10, 25 and 50 μg/kg with MON. The method was considered compliant when repeatability in terms of RSDr was lower than 20% and the recovery fell into the range 70–120% (European Commission 2016).

LOQ was defined as the lowest concentration tested that fulfilled the validation requirements of recovery and repeatability.

### Sample analysis

MON was quantified by matrix-matched calibration, i.e. by standards prepared with blank matrix extracts. Matrix-matched calibration lines were prepared in bread, pasta, wheat flour and corn flour. The concentration levels of the calibration matrix standards ranged from 5 to 100 ng/mL. Positive samples were confirmed by standard addition. The addition level was 25 or 100 μg/kg, depending on the value obtained by matrix-matched calibration.

MON was identified by similarity of retention time and the transition 97 > 41.3 on the LC-MS/MS instrument and confirmed on the LC-Orbitrap-MS instrument with the exact mass 96.9931. The criteria used to ensure the correct identification of MON were the following: (a) the retention time of the analyte in the sample extract matched that in spiked sample extract within a deviation of ±0.1 min and (b) mass accuracy ± 5 ppm (European Commission 2016).

A blank sample and a blank sample spiked at 25 μg/kg with MON were included in every batch of analysis as quality control.

## Results and discussion

### Optimisation of the chromatography

**Fig. 2:**
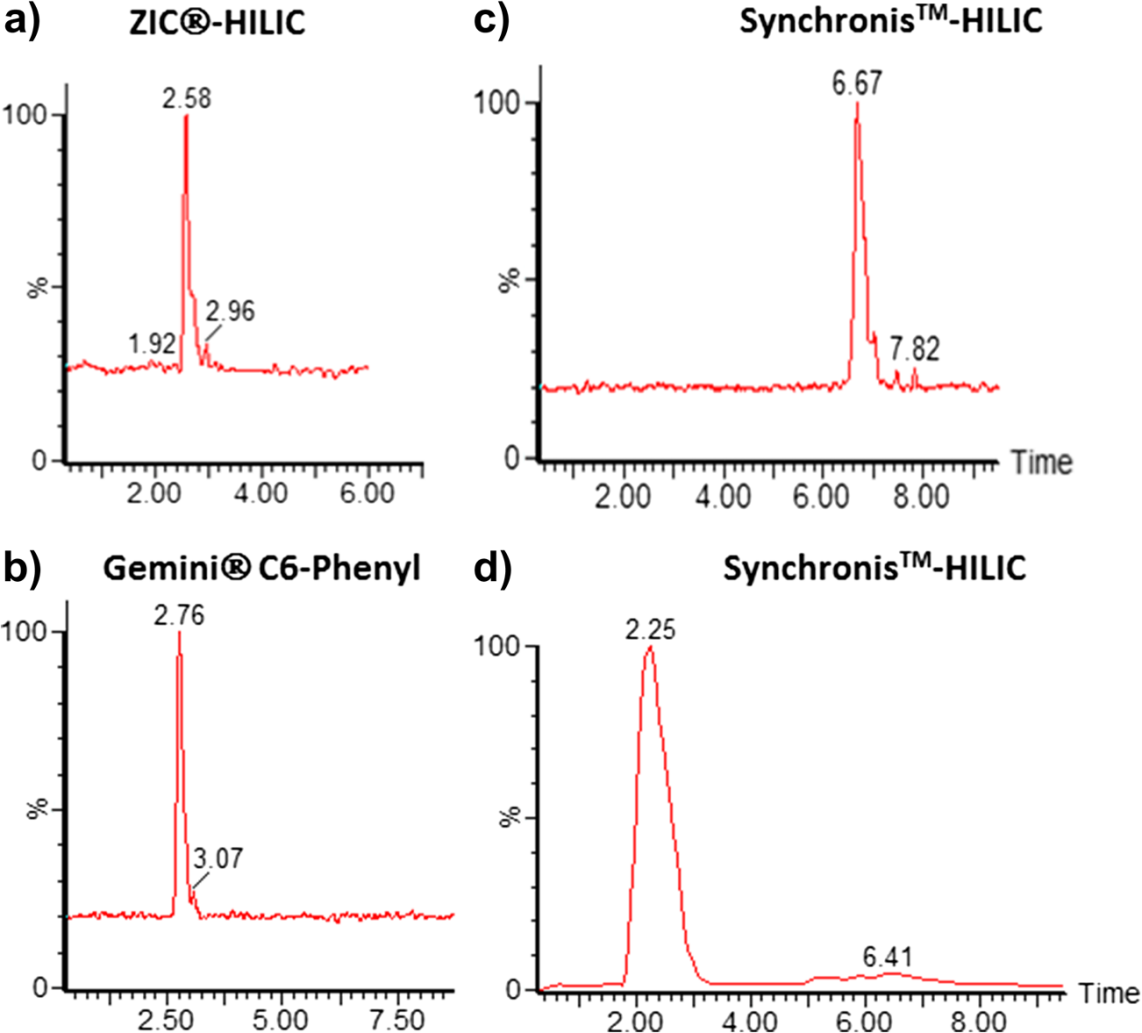
Optimization of the chromatographic separation (transition monitored 97 > 41.3), standard of 100 ng/mL in acetonitrile/water (9:1). (**a**) ZIC®-HILIC column, (**b**) Gemini ®C6-Phenyl column; (**c**) Synchronis™ column; mobile phase A water and B methanol with 1% formic acid; (**d**) Synchronis™ column mobile phase A water with ammonium formate pH 6.h and B acetonitrile

MON is polar, low-molecular-weight molecule, with ionic character, thus requiring special attention when developing a suitable chromatographic separation method. In addition, its separation from potential interfering matrix co-extractants is crucial for an accurate quantitative analysis. In previous studies, the use of different types of chromatographic columns has been reported. In the study here presented, the performance of the three chromatographic columns, Sequant® ZIC®-HILIC, Synchronis™-HILIC and Gemini® C6-phenyl, as described elsewhere (Lim et al. 2015; Scarpino et al. 2013; von Bargen et al. 2012), was assessed. Both HILIC columns are of zwitterion-type. Chromatography was carried out using water as mobile phase A and methanol with 1% formic acid as mobile phase B, with a flow of 0.2 mL/min and under isocratic conditions with 40% B for the HILIC columns and 5% B for C6-phenyl column, as described in the cited manuscripts. Figure 2 depicts the retention of a standard of 100 ng/mL of MON on these three columns. MON was more strongly retained on the Synchronis™-HILIC column than on the other two tested columns. This observation differs from that by von Bargen et al. (2012), who found similar retention of MON on a Gemini® C6-phenyl column and a Synchronis™-HILIC column, but with better peak shape.

In HILIC applications, acetonitrile (ACN) is the preferred solvent to use. Methanol, as protic solvent, could affect the stability of HILIC columns when running a large series of samples (Bernal et al. 2011). Therefore, the final mobile phase and gradient were adjusted using acetonitrile as organic solvent.

### Optimisation of the extraction procedure

Table 1 shows the recoveries obtained with the different sample treatment protocols. In a first step, bread samples spiked with MON were extracted with (84:16, v/v) acetonitrile/water and cleaned up with SAX-Strata or MycoSep® 240 MON columns, with and without evaporation. The extraction yields using acidic conditions (1% of acetic or formic acid) or QuEChERS were also determined. Additional tests were conducted without clean-up step to assess the benefits of purification protocols.

**Table 1:** Recoveries of different extraction procedures on samples of bread spiked with 100 μg/kg of moniliformin, calculated on a bread matrix-matched reference standard (MMRS)

Extraction	Clean-up	Evaporation	^a^Recovery (%)
^a^ACN/water (84:16, v/v)	No	No	69%
^a^ACN/water (84:16, v/v)	SAX	Dryness	0%
^a^ACN/water (84:16, v/v)	MycoSep® 240 MON	No	55%
^a^ACN/water (84:16, v/v)	MycoSep® 240 MON	0.5 mL	20%
^a^ACN/water (84:16, v/v)	MycoSep® 240 MON	Dryness	25%
^a^ACN/water (84:16, v/v) + 1% acetic acid	MycoSep® 240 MON	0.5 mL	30%
^a^ACN/water (84:16, v/v) + 1% formic acid	MycoSep® 240 MON	0.5 mL	5%
^a^QuEChERS	MycoSep® 240 MON	0.5 mL	27%
^b^ACN/water (50:50, v/v)	No	No	85%
^b^100% water	No	No	102%

^a^Spiked with 100 μg/kg of MON

^b^Spiked with 50 μg/kg of MON

The results of the extraction experiments, as compiled in Table 1, allow several conclusions to be drawn. Firstly, the clean-up of MON standard solutions using MycoSep® 240 MON showed better results than clean-up with SAX-Strata columns. In addition, protocols using MycoSep® 240 MON were much easier and faster to conduct than those applying SAX-Strata columns.

Secondly, when comparing recoveries of sample extracts after extraction with (~50%) and without clean-up (~70%), MON seems to be retained on the clean-up column, together with other matrix interferences. Furthermore, part of the aqueous phase containing MON might be embedded with the matrix on the column, not being recovered after the centrifugation step. So far, MycoSep® columns were successfully applied to corn but not to bread. As a matter of fact, the performance of MycoSep® 240 MON columns was assessed only with standards solution, without matrix interferences, resulting in recoveries around 60%. Therefore, this clean-up step was discarded.

Thirdly, the evaporation of the extract to dryness after MycoSep® 240 MON clean-up resulted in losses and reduced the mycotoxin content to only 25%. This was also highlighted by Scarpino et al. (2013), who found losses up to 40% for MON in maize extracts. Furthermore, MON was completely lost with longer evaporation times, as well as when evaporating HCl in the eluate from SAX-Strata SPE columns.

In addition, acidic conditions using formic acid provided lower recoveries than the extraction without acid or with acetic acid, and the application of QuEChERS method was not successful. Upon phase separation of the acetonitrile–water mixture, induced by the addition of salts, the highly polar MON is extracted with the water phase instead with required organic phase.

Since MON is highly water-soluble, the yield of extraction might improve by increasing the amount of water in the extraction solvent. Consequently, extractions with 50:50 v/v acetonitrile/water and with pure water were performed. As shown in Table 1, the best recoveries were obtained with 100% water. However, this protocol entailed the extraction of other large polar molecules from the sample that made the extract more turbid. In order to remove the turbidity and obtain a clear extract ready-to-inject on the LC-MS systems, an extra centrifuge step in Eppendorf cuvettes for small volumes (1.5 mL) at higher speed (11,500 ×*g*) was included in the protocol. Thus, the final sample preparation procedure was performed as described in the “Materials and methods” section.

### Method validation

Blank samples of bread, dry pasta, wheat and maize flour were used for method validation.

Matrix effects were assessed by comparing the response of a standard of 12.5 ng/mL prepared in solvent, which corresponds to a concentration of 50 μg/kg MON in sample, with the response of a standard of 12.5 ng/mL prepared in matrix. A decrease of signal, due to the presence of matrix, of 46, 38, 54 and 50% in the matrices bread, pasta, wheat flour and maize flour, respectively, was observed. Therefore, MON was quantified using matrix-matched calibration. Due to the different composition of maize products (waffles, corn flakes and flour) or bread samples, positive results were decided to be confirmed by standard addition.

The method gave linear response over the whole calibration range in the bread and flour matrices. However, the standard of 2.5 ng/mL in pasta had a higher response factor than the other standards (6.25, 12.5 and 25 ng/mL), which led to the conclusion that the pasta sample selected as a blank was not entirely blank and could contain either traces of MON or contamination not well-separated from the target mycotoxin. This was confirmed by the peak shape; a small shoulder peak close to the MON peak was observed only in the pasta sample spiked with the 2.5 ng/mL standard. The results of the assessment of recovery and repeatability are shown in Table 2. The criteria for recovery (70–120%) and repeatability (lower than 20%) were fulfilled for all spike levels in all targeted matrices.

**Table 2:** Method validation parameters repeatability RSD_r_ (%) and recovery (%)

Matrix	^a^Spiked at 10 μg/kg		^a^Spiked at 25 μg/kg		^a^Spiked at 50 μg/kg	
	Rec.	RSD_r_	Rec.	RSD_r_	Rec.	RSD_r_
Bread	74	4	77	4	83	5
Dry pasta	81	3	85	4	83	8
Wheat flour	77	11	80	10	78	1
Maize flour	118	4	116	6	117	4

^a^Six-fold analysis

The limit of quantification (LOQ), defined as the lowest concentration fulfilling the validation requirements of recovery and repeatability, was determined at 10 μg/kg.

### Analysis of samples

**Fig. 3:**
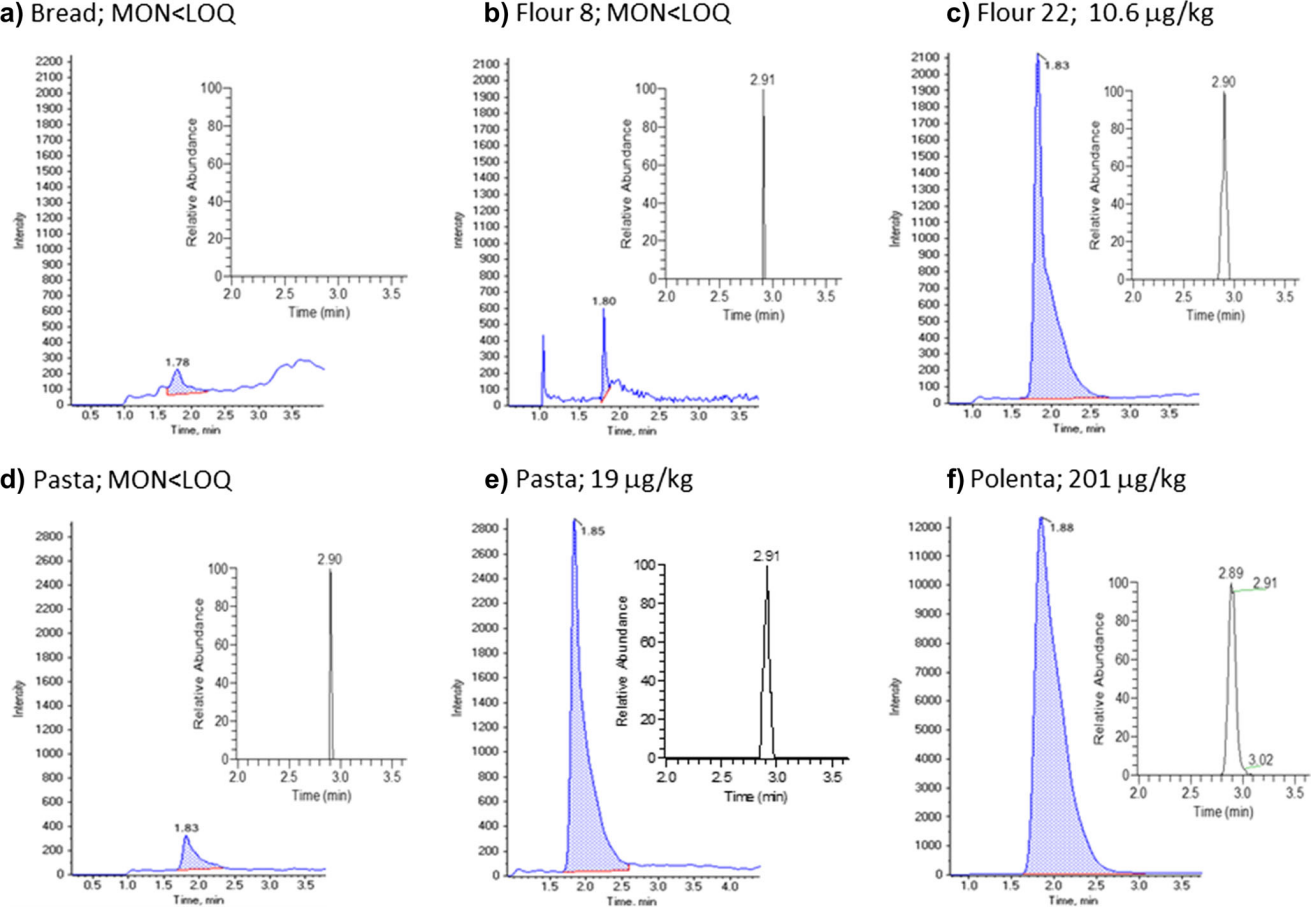
LC-MSMS with integrated area coloured and LC-Orbitrap-MS chromatograms: (**a**) bread sample; (**b**) flour sample; (**c**) flour sample; (**d**) pasta sample; (**e**) pasta sample, (**f**) polenta sample

The content of MON in the 102 samples in the present study is summarized in Table 3. Quantification of samples was conducted by LC-MS/MS. The confirmation of positive samples was carried out by LC-Orbitrap-MS. Figure 3 shows chromatograms (LC-MS/MS and HRMS) of negative and positive surveyed samples.

**Table 3:** Occurrence of moniliformin in the cereal-based samples surveyed in 2016

Food commodity	Positive samples (>^a^LOQ)	^c^Range (μg/kg)	^c^Mean (μg/kg)	^c^Median (μg/kg)
Bread (*N* = 32)	0	^b^n.a.	n.a.	n.a.
Dry pasta (*N* = 25)	7	11.1–19.0	13.3	11.8
Wheat flour (*N* = 22)	1	10.6	n.a.	n.a.
Maize-based products (*N* = 23)	8	12.3–207	111	112
Overall	16	10.6–207	62.3	18.2

^a^LOQ 10 μg/kg

^b^n.a. Not applicable

^c^Based on positive measurements

Sixteen samples out of 102, seven dry pasta samples, one wheat flour sample and eight maize-based products, showed levels of MON above the LOQ of 10 μg/kg. The maximum levels of MON (above 100 μg/kg) were found in organic maize products. More detailed information on the occurrence of MON per sample is presented in the Supplementary Information SS1.

MON was not detected in the bread samples. The wheat flour sample in which MON was detected and quantified was *Durum* wheat flour. *Durum* semolina is usually applied to prepare dry pasta. MON was detected in pasta at levels ranging from 10 to 20 μg/kg. MON was detected in two polenta samples (171, 201 μg/kg), three corn flake samples (12, 38 and 50 μg/kg), two corn waffles (24 and 207 μg/kg) and one flour sample (190 μg/kg). Seven out of the eight positive maize-based products were of organic production.

However, the total number of maize samples collected in the present study was too limited to carry out statistical evaluation on organic production and presence of MON.

Scarce information is available in literature on the occurrence on MON in cereal-based food products. The presence of MON has been mainly monitored in grains. Maize, wheat, rye, oats and triticale were reported as naturally contaminated (Sharman et al. 1991), although Norwegian wheat showed a higher susceptibility to MON contamination than oats and barley (Uhlig et al. 2004). Furthermore, MON has been reported as a common contaminant of cereals in Finland (Jestoi et al. 2004a). MON has been found in maize in different parts of the world: Poland, Australia, Canada, Germany, US and New Zeeland at very high levels, up to 131 mg/kg (Scarpino et al. 2013). In these surveys, the samples before cleaning were hand-selected in order to collect visibly *Fusarium*-infected kernels. Thus, high levels of MON contamination were expected. Scarpino et al. (2013) conducted a survey on MON occurrence for over a period of 4 years in maize grain from field or commercial lots and found lower levels of MON ranging from non-detected to a maximum of 2600 μg/kg.

As far as cereal-based food samples are concerned, MON was found in one corn flour at 131 μg/kg and in two wheat flour samples in Germany (von Bargen et al. 2012) at levels of 3.1 and 6.5 μg/kg, so below the LOQ of this study. The lower limit of quantification in the German publication was reached using a purification step with Strata-SAX SPE column, evaporation till dryness, followed by reconstitution in the mobile phase. That protocol did not work in this study, since MON was lost during the evaporation step, as can be seen in Table 1. Jestoi et al. (2004b) found that three flour samples (two wheat and one rye), purchased in retail stores in Finland, contained MON at concentrations of 25, 42 and 21 μg/kg, respectively. These levels are higher than the results found in this survey.

The fact that MON was not detected in the bread samples might be due to the LOQ level of the present study (10 μg/kg) or to processing, although MON has been reported not to be affected by baking (Pineda-Valdes et al. 2003).

*F. avenaceum* was disclosed to be the predominant producer of MON in *Durum* wheat (Kandler et al. 2002). MON had been found in *Durum* wheat samples up to 860 μg/kg. MON was detected in the present survey in dry pasta samples from *Durum* semolina. There is limited information on the fate of MON during food processing. As a matter of fact, MON is usually associated with semolina, the milling fraction of *Durum* wheat, implying that the removal of bran would not have a large impact on MON reduction (Tittlemier et al. 2014). However, the location of the fungi in the grain and its behaviour during milling and extrusion may have an influence on MON degradation. The translocation of MON from mycelium to endosperm in damaged kernels observed by Tittlemier et al. could explain the apparent loss of MON when preparing semolina from *Durum* grain. Castells et al. (2005) also observed reduction of MON after extrusion, which did not exceed 30%.

If the provisional TDI of 0.1 mg/kg bw/day, based on a single rodent assay (Peltonen et al. 2010), is taken as reference for exposure assessment, it can be concluded that the consumption of the maize samples surveyed for the present study would not entail any health concern for population. An adult of 60 kg and an infant of 25 kg would need to consume at least 30 kg and 13 kg of the maize waffles with MON at 207 μg/kg to exceed the provisional TDI.

In conclusion, a straightforward analytical method for the quantification of the mycotoxin moniliformin in cereal-based food was developed and successfully in-house validated down to 10 μg/kg. This method was fast and environmental-friendly since the use of organic solvents for extraction purposes was reduced and easy to implement in routine analysis. The identification criteria derived from the Commission Decision 2002/657/EEC were met by the method presented here, by combining quantitative LC-MS/MS analysis with confirmation with accurate mass by means of LC-Orbitrap-HRMS. The method was applied to a survey of 102 samples of flour, bread and pasta collected in the Netherlands and Germany. Moniliformin was detected in one *Durum* flour sample at 10.6 μg/kg, in seven pasta samples at levels between 10 and 20 μg/kg and in eight maize products at levels between 12 and 207 μg/kg. The major incidence of MON occurred in organic samples, but the number of maize samples in this study was too limited to draw sensible conclusions on this issue. The consumption of products contaminated with MON does not pose any health risk for consumers.

This type of survey is recommended to be repeated along the years to evaluate year-to-year variation from the current study.

## Electronic supplementary material

ESM 1 (DOCX 32 kb).
