# Supplementary material for: Survey of moniliformin in wheat- and corn-based products using a straightforward analytical method
Source: Mycotoxin Res. 2017 Aug 8;33(4):333–41. doi: 10.1007/s12550-017-0287-9 (PMC5644698; doi:10.1007/s12550-017-0287-9)
Supplement: Supplementary file 1 — (DOCX 32 kb). [file 12550_2017_287_MOESM1_ESM.docx]

**SUPPLEMENTARY INFORMATION to the paper entitled:**

**Survey of moniliformin in wheat and corn-based products using a straightforward analytical method**

Marta Herrera^a^, Ruud van Dam^b^, Martien Spanjer^c^, Joyce de Stoppelaar^c^, Hans Mol^b^, Monique de Nijs^b^, Patricia López^b^*,

^a^Instituto Agroalimentario de Aragón IA2 (Universidad de Zaragoza-CITA), Veterinary Faculty, 50013 Zaragoza, Spain

^b^RIKILT Wageningen University & Research, Akkermaalsbos 2, 6708 WB, Wageningen, The Netherlands

^c^NVWA – Netherlands Food and Consumer Product Safety Authority, Catharijnesingel 59, 3511 GG Utrecht, The Netherlands

*Corresponding author: patricia.lopezsanchez@wur.nl

Supplementary Information Table SS1. Detailed information on the surveyed samples

| Sample | Country of sampling^a^ |  | O/C^b^ | MON (μg/kg) |
| --- | --- | --- | --- | --- |
| Flour1 | DE | Wheat wholemeal flour | C | < 10 |
| Flour2 | DE | Wheat flour type 550 | C | < 10 |
| Flour3 | DE | Wheat flour type 405 | C | < 10 |
| Flour4 | DE | Wheat semolina | O | < 10 |
| Flour5 | DE | Multigrain baking mixture | C | < 10 |
| Flour6 | DE | Multigrain baking mixture | C | < 10 |
| Flour7 | NL | Wheat flour | C | < 10 |
| Flour8 | NL | Mixture multigrain for crepes | C | < 10 |
| Flour11 | NL | 6-grains mix | O | < 10 |
| Flour12 | NL | Chick pea flour | O | < 10 |
| Flour13 | NL | Multigrain flour | O | < 10 |
| Flour14 | NL | Spelt flour | O | < 10 |
| Flour15 | NL | Self-rising flour | C | < 10 |
| Flour16 | NL | Wheat flour | C | < 10 |
| Flour17 | NL | Multigrain mix for bread | C | < 10 |
| Flour18 | NL | Multigrain flour for bread with seeds | C | < 10 |
| Flour19 | NL | Multigrain mix for crepes | C | < 10 |
| Flour20 | NL | Wholemeal wheat flour | C | < 10 |
| Flour21 | NL | Organic amaranth flour | O | < 10 |
| Flour22 | NL | *Durum* flour | O | 10.6 |
| Flour23 | NL | *Durum* flour | O | < 10 |
| Flour24 | NL | *Durum* flour | O | < 10 |
| BBR1 | DE | Multigrain bread | C | < 10 |
| BBR3 | DE | Multigrain bread | C | < 10 |
| BBR4 | DE | Multigrain | C | < 10 |
| BBR5 | DE | Multigrain bread | O | < 10 |
| BBR6 | DE | Wholegrain bread | C | < 10 |
| BBR7 | DE | Multigrain bread | C | < 10 |
| BBR8 | DE | Wheat bread | C | < 10 |
| BBR9 | DE | Wheat bread | C | < 10 |
| BBR10 | DE | Wheat bread | C | < 10 |
| BBR11 | DE | Wheat bread | C | < 10 |
| BBR12 | DE | Wheat bread | O | < 10 |
| BBR13 | NL | Multigrain pancake | C | < 10 |
| BBR14 | NL | Dark wholegrain bread | C | < 10 |
| BBR15 | NL | White sandwich bread | C | < 10 |
| BBR16 | NL | White party bread (cut) | C | < 10 |
| BBR17 | NL | Pistolets multi grain | C | < 10 |
| BBR18 | NL | White bread | C | < 10 |
| BBR19 | NL | White bread | C | < 10 |
| BBR20 | NL | Multigrain bread | C | < 10 |
| BBR21 | NL | White fibre bread | C | < 10 |
| BBR22 | NL | Wheat bread with corn | C | < 10 |
| BBR23 | NL | Multigrain rolls | C | < 10 |
| BBR24 | NL | Organic dark multigrain bread | O | < 10 |
| BBR25 | NL | Multigrain bread | C | < 10 |
| BBR26 | NL | Hearty multigrain | C | < 10 |
| BBR27 | NL | Wholemeal spelt bread | C | < 10 |
| BBR28 | NL | White bread | C | < 10 |
| BBR29 | NL | White bread | C | < 10 |
| BBR30 | NL | White bread | C | < 10 |
| BBR31 | NL | Multigrain bread | C | < 10 |
| BBR32 | NL | Multigrain bread | C | < 10 |
| BBR33 | NL | White bread with corn |  | < 10 |
| Pasta 1 | DE | Spirals | C | 17.4 |
| Pasta 2 | DE | Farfalle | C | < 10 |
| Pasta 3 | DE | Tortiglioni | C | 19.0 |
| Pasta 4 | DE | Cannelloni | C | 10.4 |
| Pasta 5 | DE | Spelt spaghetti | O | < 10 |
| Pasta 6 | DE | Fork spaghetti | C | < 10 |
| Pasta 7 | DE | Spaghetti | C | < 10 |
| Pasta 8 | DE | Penne rigate | C | < 10 |
| Pasta 9 | DE | Macaroni | C | < 10 |
| Pasta 10 | DE | Tomato Basilikum Totiglioni | C | < 10 |
| Pasta 11 | NL | Italian spaghetti from durum wheat | C | < 10 |
| Pasta 12 | NL | Durum wholemeal wheat cornetti | O | < 10 |
| Pasta 13 | NL | Emmer wholemeal spaghetti | O | < 10 |
| Pasta 14 | NL | Spaghetti | C | < 10 |
| Pasta 15 | NL | Spaghetti | C | 11.8 |
| Pasta 16 | NL | Bio organic spaghetti | O | < 10 |
| Pasta 17 | NL | Fusili tricolori | C | < 10 |
| Pasta 18 | NL | Wholegrain spelt penne | C | < 10 |
| Pasta 19 | NL | Macaroni | C | < 10 |
| Pasta 20 | NL | Farfalle | C | < 10 |
| Pasta 21 | NL | Spaghetti | C | 11.6 |
| Pasta 22 | NL | Bio Penne Rigate | O | < 10 |
| Pasta 23 | NL | Macaroni | C | 11.8 |
| Pasta 24 | NL | Taglatielles | C | < 10 |
| Pasta 25 | NL | Pipe rigate | C | 11.1 |
| Maize 01 | NL | Corn waffles | O | < 10 |
| Maize 02 | NL | Corn flakes | C | < 10 |
| Maize 03 | NL | Corn flakes | C | < 10 |
| Maize 04 | NL | Corn flakes | C | < 10 |
| Maize 05 | NL | Corn flakes | C | < 10 |
| Maize 06 | NL | Tortilla corn chips salted | O | < 10 |
| Maize 07 | NL | Polenta | O | < 10 |
| Maize 08 | NL | Corn flakes | C | < 10 |
| Maize 09 | NL | Corn waffles | O | < 10 |
| Maize 10 | NL | Corn waffles | O | 24.2 |
| Maize 11 | NL | Corn flakes | C | 37.8 |
| Maize 12 | NL | Corn flakes | C | < 10 |
| Maize 13 | NL | Corn flour | O | < 10 |
| Maize 14 | NL | Corn flour | C | < 10 |
| Maize 15 | NL | Corn flour | C | < 10 |
| Maize 16 | NL | Corn waffles | O | < 10 |
| Maize 17 | NL | Corn flakes | O | 12.3 |
| Maize 18 | NL | Corn flakes | O | 50.4 |
| Maize 19 | NL | Corn waffles | O | < 10 |
| Maize 20 | NL | Corn flour | O | 190 |
| Maize 21 | NL | Polenta | O | 171 |
| Maize 22 | NL | Polenta | O | 201 |
| Maize 23 | NL | Corn waffles | O | 207 |

^a^Country of sampling: NL: Netherlands; DE: Germany

^b^O/C: organic/conventional
